# Pathophysiology of Pre-Eclampsia—Two Theories of the Development of the Disease

**DOI:** 10.3390/ijms25010307

**Published:** 2023-12-25

**Authors:** Jakub Kornacki, Olga Olejniczak, Rafał Sibiak, Paweł Gutaj, Ewa Wender-Ożegowska

**Affiliations:** 1 Department of Reproduction, Chair of Reproduction and Perinatal Medicine, Poznan University of Medical Sciences, 61-701 Poznan, Poland; olejniczakolgamaria@gmail.com (O.O.); rafal.sibiak@student.ump.edu.pl (R.S.); pawelgutaj@ump.edu.pl (P.G.); ewozegow@ump.edu.pl (E.W.-O.); 2 Department of Histology and Embryology, Poznan University of Medical Sciences, 60-701 Poznan, Poland; 3 Doctoral School, Poznan University of Medical Sciences, 60-701 Poznan, Poland

**Keywords:** pre-eclampsia, placenta, anti-angiogenic factors, placental growth factor, cardiovascular impairment, echocardiography

## Abstract

Pre-eclampsia (PE) continues to be a leading cause of maternal and fetal mortality and morbidity. While substantial progress has been made in understanding the pathomechanisms of PE, the pathophysiology of the disease is still not fully understood. While the “two-stage model” of the development of PE is the most widely accepted theory, stating that the placenta is the main source of the disease, there are some other pathophysiological models of PE. Among these other theories, the one considering heart dysfunction as serving as the primary cause of PE seems to be gaining increasing prominence. In this review, we aim to elucidate these two divergent concepts concerning the development of PE. Despite some differences in their proposed pathomechanisms, both theories share vital pathophysiological elements in common. A central and critical component in both models is impaired placental perfusion, which appears to be a crucial phenomenon in PE. A comprehensive understanding of the different pathomechanisms involved in PE may be helpful in clinical practice, prompting a more individual approach to care of patients with PE.

## 1. Introduction

Despite significant advances in the field of maternal–fetal medicine in recent years, pre-eclampsia (PE) remains a leading cause of maternal and fetal mortality, particularly in less developed countries [1,2]. However, substantial clinical progress in the prediction and prophylaxis of PE has led to a gradual change in this field, which should contribute to a significant reduction in its prevalence in the future, especially in early-onset PE, primarily in well-developed countries [3]. This progress has been made possible through global research efforts and precise clinical observations. From a research perspective, gathering knowledge about the intricate etiology and pathophysiology of PE has been the most important focus. Consequently, we now possess a wealth of knowledge about the pathogenesis of the disease, including the sequential stages of its development. This, in turn, has enabled clinicians to implement effective screening methods for PE at various stages of pregnancy, such as ultrasound Doppler and biochemical tests [4].

In spite of such great achievements in understanding the pathophysiology of PE, there are still some doubts and controversies. This is mainly because PE is a heterogeneous disease, with many etiological factors and different clinical presentations. These facts suggest the existence of more than one pathogenesis of the disease.

Currently, the prevailing and most plausible theory regarding the development of PE is the two-stage theory. In this model, the first stage of the disease involves impaired placentation and reduced placental perfusion, while the second stage encompasses general maternal endothelial damage and dysfunction [5,6,7]. This theory offers a comprehensive explanation, particularly for early-onset PE, which is characterized by placental insufficiency and fetal growth restriction (FGR). However, late-onset PE, which is rarely associated with significant placental dysfunction, may have a slightly different pathophysiological basis.

The second theory of the development of PE is a cardiogenic one. According to this model, the primary cause of the disease is a pre-existing cardiac dysfunction, which is triggered or exacerbated by pregnancy. This cardiac dysfunction subsequently leads to reduced placental perfusion and the characteristic clinical consequences observed in PE [8].

## 2. Methods

To compile this review, we conducted a comprehensive search of the PubMed database for relevant references up to October 2023 using the following terms: “pathophysiology preeclampsia”, “pathomechanism preeclampsia”, “two stage model preeclampsia”, “trophoblast invasion preeclampsia”, “endothelial dysfunction preeclampsia”, “cardiovascular system preeclampsia”, “echocardiography preeclampsia”, “cardiovascular system early-onset preeclampsia late–onset preeclampsia”. Additionally, we reviewed the bibliographies of the included articles to identify any further relevant publications, which aligned with the objectives of this paper. Subsequently, we examined the full texts of each article included in this review. We finally selected only the articles written in English, which could represent a potential limitation of our study.

## 3. The First Theory of the Development of PE—PE as a Placental Pathology

### 3.1. The First Stage of PE—Impaired Placental Perfusion

#### 3.1.1. Impaired Trophoblast Invasion and Abnormal Remodeling of Spiral Arteries

The prevailing and widely accepted theory for the development of PE is the “two-stage model”. In this model, the primary cause of PE is the abnormal development of the placenta associated with a subsequent reduction in placental perfusion. As per this theory, the first stage of the development of PE involves reduced placental perfusion, followed by a generalized maternal endothelial dysfunction [9].

According to this theory, the initial reduction in placental perfusion is mainly caused by disruptions in the process of placental development, known as placentation. This disturbance arises from impaired trophoblast invasion into the spiral arteries during the first half of pregnancy [10,11].

**Figure 1:**
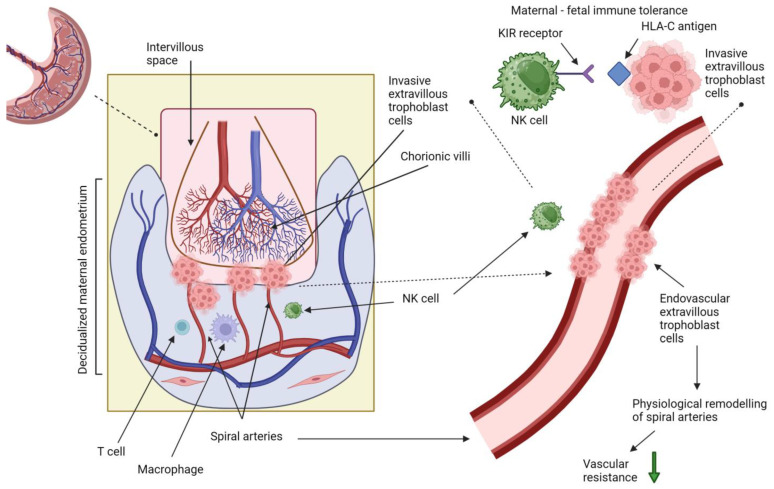
The physiological process of trophoblast invasion and remodeling of spiral arteries. Own authorship, created with Biorender.com (accessed on 23 November 2023).

In the physiological course of the first trimester of pregnancy, extravillous trophoblasts infiltrate the lumen and walls of spiral arteries, as shown in Figure 1. The consequence of this invasion is a remodeling of the spiral arteries’ walls, including the loss of a smooth muscle and elastic lamina, as well as a temporary replacement of endothelial cells with trophoblast cells [12,13,14,15]. This physiological remodeling of spiral arteries converts high-resistance arterioles into low-resistance vein-like vessels [14,15]. This transformation ensures a stable and adequate perfusion of the pregnant uterus, including the placenta.

**Figure 2:**
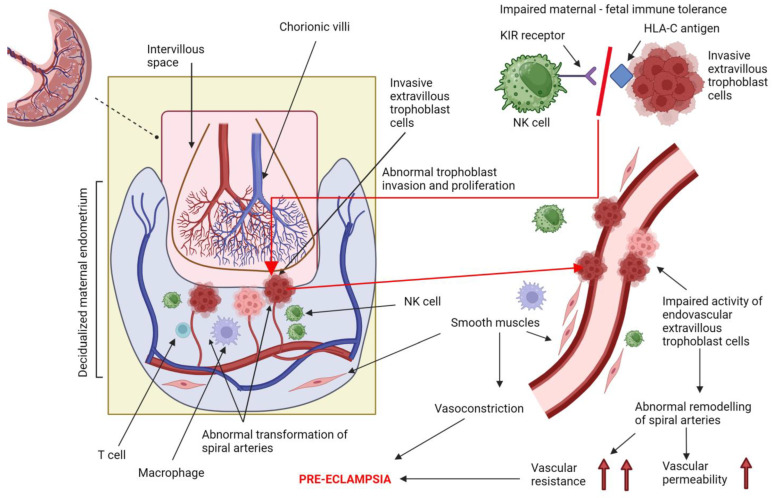
Abnormal placentation as a result of impaired trophoblast invasion. Own authorship, created with Biorender.com (accessed on 23 November 2023).

It was first discovered in 1967 that PE is associated with abnormal placentation as a result of impaired trophoblast invasion [16]. Therefore, in contrast to normal pregnancies, women who develop PE experience an impaired remodeling of spiral artery walls, which fails to provide optimal placental development, potentially leading to reduced placental perfusion from the first half of pregnancy [17], as shown in Figure 2. Subsequent research demonstrated that the consequence of the impaired remodeling of spiral arteries is more detectable in the quality of placental perfusion, leading to a more pulsatile and higher pressure flow rather than reduced perfusion [18,19].

The physiological process of spiral artery remodeling is complicated and involves different interactions among maternal uterine immune cells and embryonic cells, particularly trophoblasts [12,20]. The maternal cells involved in this process mainly include decidual natural killer (NK) cells, T cells and macrophages [12,20]. The interactions between extravillous trophoblast (EVT) human leukocyte antigens (HLA) and both NK and T cells appear to be crucial in promoting maternal–fetal immune tolerance and facilitating the proper process of placentation, including trophoblast invasion into spiral arteries [21,22,23,24]. In this unique maternal–fetal “dialog”, the appropriate recognition of trophoblast human leukocyte antigen-C (HLA-C) by NK cells expressing killer cell immunoglobulin-like receptor (KIR) seems to be especially important [22,25,26].

Moreover, the role of the immune system in the phenomenon of placentation may also explain the reduced risk of placental complications in subsequent pregnancies.

#### 3.1.2. Different Causes of Impaired Placental Perfusion

Impaired placental perfusion, recognized as the first step in the development of PE, may have etiologies other than impaired trophoblast invasion. The possible causes of impaired placental perfusion may include (1) primary pathologies of the vessel walls, characteristic for some autoimmune disorders, such as lupus or scleroderma or diabetes; (2) primary dyslipidemia affecting the quality of vascular perfusion; (3) hypercoagulability or thrombophilia, especially the anti-phospholipid syndrome; (4) obesity, which is associated with inflammation of the vessel walls; (5) enlarged, big placentas, which are associated with increased risk of hypoxia; (6) chronic hypertension; and (7) acute atherosis [17].

##### Acute Atherosis

This phenomenon is mainly associated with PE and especially concerns the distal decidual segments of non-remodeled spiral arteries [27,28]. It is characterized by the presence of foam cells in the arterial walls of the decidua, probably as a reaction to inflammatory, immunogenetic and hemodynamic triggers [21,29,30]. Dyslipidemia, which is much more evident in patients who develop PE than in pregnant women with physiological pregnancy, may further predispose to it [31]. Acute atherosis may further impair placental perfusion in women with PE.

##### Other Pathologies, which May Impair Placental Perfusion

All the above-mentioned pathologies or maternal morbidities may negatively influence placental perfusion even in cases of adequate placentation. They may theoretically occur in each period of pregnancy and may lead both to early- and late-onset PE. The two risk factors more frequently associated with late-onset PE are diabetes and enlarged placenta [32]. In the case of the latter, big placentas may be more prone to relative hypoxia being at the same time the source of increased number of anti-angiogenic factors compared to normal placentas.

All the pathologies listed, which impair placental perfusion, are very important risk factors for PE [10].

### 3.2. The Link between the First and the Second Stage of PE

The most probable pathophysiological connection between the first stage of PE, characterized by impaired placental perfusion, and the subsequent second stage, marked by maternal endothelial dysfunction, is the increased production of anti-angiogenic factors by poorly perfused placentas in women who later develop PE [7]. These anti-angiogenic factors, if produced in excess, have a negative influence both on the integrity and function of the maternal endothelium, ultimately leading to its damage and dysfunction [5,33].

The two main anti-angiogenic factors produced in the placenta, which are involved in the pathophysiology of PE, are soluble fms-like tyrosine kinase 1 (sFlt1) and soluble endoglin (sEng). Of these, sFlt-1 has attracted more attention and is now used as a clinical marker for PE in daily practice.

SFlt-1 is a soluble form of the vascular endothelial growth factor (VEGF) receptor 1. It inhibits the proangiogenic activity of VEGF, including its protective function on the endothelium. It has the capacity to bind both the placental growth factor (PlGF) and VEGF, resulting in a negative impact on placenta development and on maternal endothelium [34,35,36].

The increased production of sFlt-1 in the placenta of women who develop PE is probably a consequence of the previously mentioned placental hypoxia [37]. It has been observed that in hypoxic conditions, there is an increased production of sFlt-1, which represents a shorter splice variant of the Flt-1 receptor [38]. This shift toward overproduction of sFlt-1 rather than full-length membrane receptor (Flt-1) may result from decreased activity of an oxygen-sensing protein (oxygenase) called Jumonji domain-containing protein 6 (JMJD6) [39].

SEng, another anti-angiogenic factor, may contribute to endothelial injury similarly to sFlt-1 [7,40]. The probable mechanism of action of sEng involves its binding to transforming growth factor-beta 1 (TGF-β1), which can further inhibit the production of nitric oxide (NO) [7,41,42].

### 3.3. The Second Stage of PE—Maternal Endothelial Injury and Dysfunction

Maternal endothelial injury and dysfunction are responsible for the manifestation of most clinical symptoms characteristic of PE, including edema, thrombocytopenia, neurological complications, visual disturbances and proteinuria [5,17].

**Figure 3:**
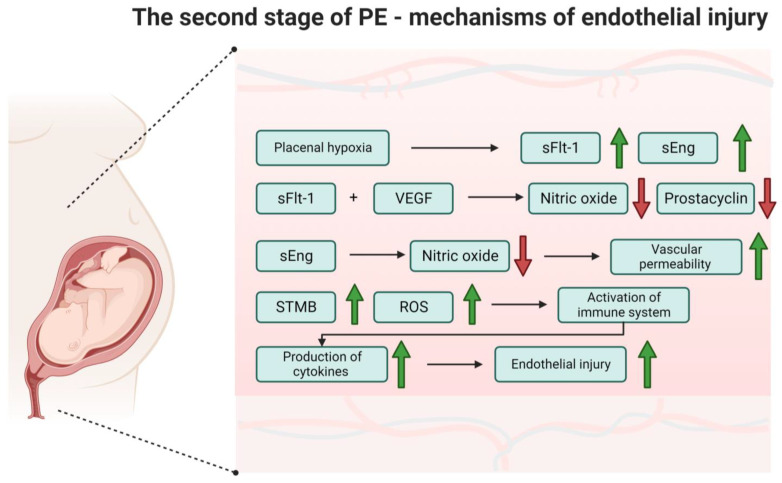
The second stage of pre-eclampsia—mechanisms of endothelial injury. Abbreviations: ROS—reactive oxygen species, sEng—soluble endoglin, sFlt-1—soluble fms-like tyrosine kinase 1, STMB—syncytiotrophoblast microparticles, VEGF—vascular endothelial growth factor. Own authorship, created with Biorender.com (accessed on 23 November 2023).

The primary mechanism responsible for endothelial dysfunction in PE appears to be the anti-angiogenic activity of sFlt-1 and sEng, which are overproduced in women who develop PE, as shown in Figure 3.

The anti-angiogenic effect of sFlt-1 is a consequence of its binding to VEGF. One of the roles of VEGF is vascular and endothelial protection, which includes promoting endothelial cell survival and stimulating the production of nitric oxide and prostacyclin [33]. Consequently, the presence of excess sFlt-1 can impair VEGF’s protective role. Furthermore, the anti-angiogenic effect may be intensified by sEng activity, which may cause a reduction in the production of NO by endothelial cells and an increase in vascular permeability [43].

Another likely contributor to the maternal endothelial injury and dysfunction characteristic of PE is the activation of the maternal immune system [44,45,46]. This activation is characterized by an increased production of cytokines by different immune cells [44,45,46]. The activation of the immune system in PE may result from the increased shedding of syncytiotrophoblast microparticles (STMB) from a hypoxic placenta into maternal circulation and/or the increased production of reactive oxygen species (ROS) in the placenta [44,45,46].

The different markers of endothelial injury and dysfunction may be examined. They include (1) serum concentrations of markers of endothelial activation, such as vascular cell adhesion molecule-1 (VCAM-1) and selectins, especially E-selectin; (2) serum levels of markers of endothelial glycocalyx (EG) degradation, such as hyaluronan (HA) and syndecan-1 (SDC-1); (3) the concentration of endothelin-1 (ET-1); and (4) the level of circulating endothelial cells (CECs) and circulating endothelial progenitor cells (CEPCs) [6]. Flow-mediated dilation (FMD) stands as the gold standard for in vivo, non-invasive assessment of endothelial function [47,48,49].

In numerous studies, patients with PE have been shown to exhibit increased concentrations of the aforementioned markers of endothelial dysfunction compared to healthy pregnant women [33,50,51,52,53,54,55,56,57,58,59,60].

Given the complex and heterogenic pathophysiology of PE, along with its diverse clinical manifestations, an interesting area of investigation is the extent of endothelial dysfunction in the two primary forms of PE: early-onset PE (EOP) and late-onset PE (LOP). To date, the results in this regard have been somewhat conflicting. While our studies [5,33] and the study by Rios et al. [61] found no difference between EOP and LOP, Weissgerber et al. [62] reported a higher degree of endothelial damage in women with EOP compared to patients with LOP.

The sequential involvement of the placenta and then the maternal endothelium in the pathophysiology of PE, as suggested by this model, appears to make the “two-stage theory” more applicable to EOP than LOP. However, the comparable degree of endothelial dysfunction in both types of PE seems to support the use of this model to explain both forms of the condition.

Currently, the fundamental elements of this model for the development of PE have been effectively integrated into daily clinical practice. The best example is the assessment of the sFlt-1/ PlGF ratio in the management of patients with PE [63]. The introduction of this method into daily practice has successfully streamlined the hospitalization or delivery-related decision-making process for women with both early- and late-onset PE.

## 4. The Second Theory of the Development of PE—PE as a Result of Maladaptation of the Maternal Cardiovascular System

### 4.1. PE and Cardiovascular Diseases—Common Risk Factors

#### 4.1.1. Common Risk Factors for Cardiovascular Diseases and for PE—Current State of Knowledge

Recent studies have suggested that cardiovascular diseases (CVD) and PE share many risk factors [64]. The recognized risk factors for PE, according to the American College of Obstetricians and Gynecologists (ACOG), include nulliparity, multiple gestations, a history of PE in a previous pregnancy, chronic hypertension, pre-gestational diabetes, gestational diabetes, thrombophilia, systemic lupus erythematosus, pre-pregnancy body mass index greater than 30, anti-phospholipid syndrome, maternal age 35 years or older, kidney disease, assisted reproductive technology and obstructive sleep apnea [65].

The risk factors for CVD, as identified by the American Heart Association [66] and the American Stroke Association [67], overlap with those for PE, with the exception of factors specific to pregnancy.

Giannakou et al. [68] identified the risk factors most strongly associated with PE. These include, among others, chronic kidney disease, polycystic ovarian syndrome (PCOS), oocyte donation, obesity and primiparity.

Women with PCOS have an increased risk of developing PE compared to controls [69]. Insulin resistance and concomitant hyperglycemia, often present in patients with PCOS and obesity, may predispose individuals to hypertension by causing endothelial function impairment [69]. PCOS also increases cardiovascular risk outside of pregnancy [70,71].

#### 4.1.2. The Imbalance in Lipid Metabolism—Possible Cause of Endothelial Dysfunction and Therefore PE

**Figure 4:**
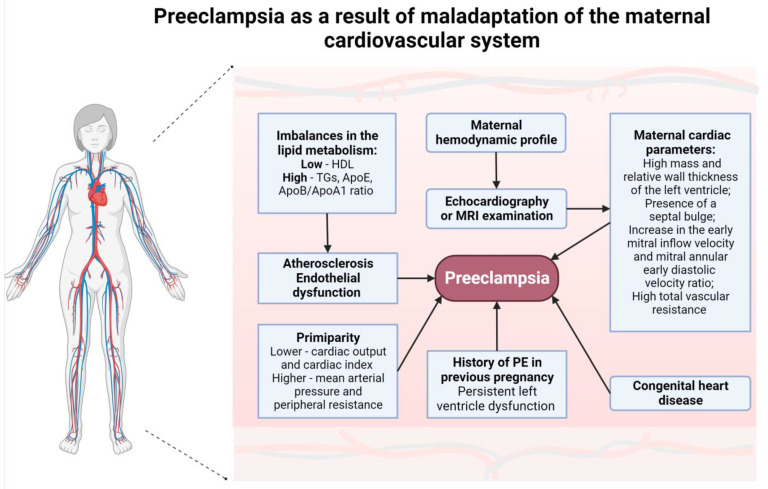
Pre-eclampsia as a result of maladaptation of the maternal cardiovascular system. Abbreviations: ApoA1—apolipoprotein A1, ApoB—apolipoprotein B, ApoE—apolipoprotein E, HDL—high-density lipoprotein, MRI—magnetic resonance imaging, PE—pre-eclampsia, TGs—triglycerides. Own authorship, created with Biorender.com (accessed on 18 December 2023).

Low levels of high-density lipoprotein (HDL) cholesterol, elevated triglycerides (TGs), Apolipoprotein E (ApoE) concentrations and the Apolipoprotein B to Apolipoprotein A1 (ApoB/ApoA1) ratio have been associated with a higher prevalence of hypertension and PE, as shown in Figure 4 [72].

High TGs and low HDL-C are characteristic parameters of a cardio-metabolic profile known as atherogenic dyslipidemia, which correlates with the metabolic syndrome, insulin resistance and diabetes. Based on this finding, it can be concluded that the TG/HDL-C ratio is associated with an increased cardiovascular risk in general populations [73].

The ApoE gene has common alleles, which encode isoforms reliably associated with high triglyceride and very-low-density lipoprotein (VLDL) levels. This strongly suggests that APOE gene polymorphisms are probably responsible for the increased risk of developing cardiovascular diseases [74].

ApoB is the major protein in very-low-density (VLDL), intermediate-density (IDL) and low-density lipoproteins (LDL). ApoA-I is the major protein in high-density lipoprotein (HDL). Both apolipoproteins play a significant role in lipid transport and the process of atherosclerosis and its complications. The apoB/apoA-1 ratio indicates the balance between atherogenic and anti-atherogenic particles, and an increased value of this parameter is associated with a higher probability of developing CVD [74].

Imbalances in the levels of metabolites related to oxidative stress and lipid metabolism, in which apolipoproteins are involved, are associated with the process of arteriosclerosis. This can lead to endothelial dysfunction, a characteristic feature of PE [75].

#### 4.1.3. Good Condition of the Maternal Cardiovascular System Is a Guarantee of an Uncomplicated Pregnancy

In their study, Romundstad et al. [76] proved that the cardiovascular risk factors present before pregnancy, complicated by hypertensive disorders, are more likely to contribute to cardiovascular risk than hypertensive pregnancy itself. This suggests that the condition of the mother’s cardiovascular system may play a significant role in the development of PE or gestational hypertension, and consequently, cardiovascular diseases later in life.

#### 4.1.4. Smoking and the Risk of Developing Pre-Eclampsia—Unexpected Correlation

While all the above-mentioned risk factors are strongly associated with the risk of developing cardiovascular disease in non-pregnant adults [76,77], there is one exception. Epidemiological studies have paradoxically shown that smoking during pregnancy is associated with a reduced risk of PE. The biological mechanism through which smoking exerts a protective effect against PE remains unknown [78]. One possible explanation for this unexpected effect of smoking, a potent risk factor for CVD [79], is that the carbon monoxide from cigarette smoke inhibits the release of two potent anti-angiogenic factors associated with PE, namely sFlt- 1 and sEng, from endothelial cells and the placenta.

#### 4.1.5. Common Risk Factors for CVD and PE—A Step toward Understanding the Pathophysiology of PE

It is difficult to definitively determine whether PE is an independent predictor of CVD development or whether the primary causes of CVD in patients with PE are adverse cardiovascular profiles associated with other factors, such as obesity and hypertension. The strong association between the risk factors for CVD and those associated with PE prompts us to consider whether there is a common basis in the pathophysiology of both conditions. Pregnancy appears to serve as a kind of metabolic and vascular stress test for the maternal cardiovascular system [80]. When the patient’s cardiovascular system is in a poor condition, “the test is failed”, and this impairment becomes evident in the development of de novo hypertensive disorders during pregnancy, including PE, or an increased likelihood of cardiovascular complications after pregnancy. The occurrence of PE in past pregnancies also raises the risk of recurrence in a subsequent pregnancy, possibly due to inadequate regeneration of the cardiovascular system after a pregnancy complicated by PE [80,81].

### 4.2. Cardiovascular Impairment Associated with Pre-Eclampsia

#### 4.2.1. Physiology of Cardiovascular System during Pregnancy

During the course of pregnancy, the maternal circulatory system undergoes significant changes. The total blood volume of a pregnant woman increases by approximately 40% by the end of gestation. This alteration includes a 20–30% increase in erythrocytic mass and a 45–55% increase in plasma volume [82].

As a result, there is an increase in pre-load, leading to a rise in venous return, as evidenced by the enlargement of left atrial volumes. Simultaneously, the after-load is reduced due to a decrease in total vascular resistance and mean arterial pressure. The cardiac output, defined as the product of stroke volume (SV) and heart rate (HR), increases throughout pregnancy, reaching its peak at 22–24 weeks of gestation [83].

These physiological hemodynamic changes induce a compensatory response from the maternal heart, primarily manifested in the remodeling of the left ventricle (LV), such as eccentric hypertrophy in LV and an increase in LV mass compared to the pre-gestation values [84].

In uncomplicated pregnancies, the myocardial structure and function return to a normalized state after delivery, similar to or matching the condition before gestation [85,86].

Thanks to these hemodynamic changes characterized by increased intravascular volume and cardiac output, an adequate uteroplacental blood flow is ensured, which allows the optimal flow of oxygen and nutrients to the placenta, and therefore, to the growing fetus.

#### 4.2.2. Congenital Heart Disease as a Basis for PE

The proper functioning of the maternal cardiovascular system and the uncomplicated course of pregnancy are interdependent. This correlation is indirectly substantiated by several studies demonstrating that women with congenital heart disease (CHD) are more likely to develop PE or FGR than those without comorbidities.

A large retrospective study comparing pregnancy outcomes between women with and without CHD has been carried out. The study included 3,642,041 patients admitted to hospitals in California for delivery, comprising 3,638,590 women without CHD diagnosis, 3189 women with non-complex CHD and 262 women with complex CHD. Complex CDH included endocardial cushion defects, hypoplastic left heart syndrome, tetralogy of Fallot, transposition of the great arteries, truncus arteriosus and univentricular heart defects, while other types of congenital heart defects were classified as non-complex CHD. One of the main findings of the study indicated a strong association between non-complex CHD and an increased risk of developing PE [87].

This finding is consistent with the results of a study of 714 women with CHD performed by Drenthen et al. [88], which demonstrated that the most significant obstetric complications during pregnancy reported in 12% of participants were hypertension-related disorders (HRD), including PE, diagnosed in 4% of pregnancies. HRD conditions were diagnosed predominantly in patients with uncorrected CHD.

Furthermore, another study revealed a higher prevalence of PE in women with uncorrected atrial septal defects (ASD) compared to the general population, although this correlation was not observed in women who underwent ASD correction surgery before pregnancy [89].

These findings collectively suggest that the pre-pregnancy condition of the maternal cardiovascular system plays a critical role in the development of PE.

This hypothesis is supported by several studies, which have shown that some abnormal features in cardiac parameters occur before clinical symptoms of PE.

#### 4.2.3. Features in Echocardiography Associated with PE

Echocardiographic analysis of the maternal cardiac structure and function during mid-gestation can reveal differences between women who will subsequently develop PE and those who have uncomplicated pregnancies.

A study involving 526 high-risk primigravidas with bilateral notching of the uterine artery Doppler proved that echocardiography performed at mid-gestation might help identify patients who would subsequently develop maternal and fetal complications. Left ventricular morphological parameters (high relative wall thickness of the LV and high LV mass) were significantly different in pregnancies, which later developed complications. High total vascular resistance (TVR) was identified as an independent predictor of subsequent development of hypertensive disorders in pregnancy [90].

Another study by Melchiorre et al. [91] also reported increased LV mass in women diagnosed with PE at term. However, this cardiac parameter seemed to remain unchanged when measured at mid-gestation, before the onset of the disease. The signs of LV geometric remodeling in pregnancies complicated by PE were noticeable both before the appearance of clinical symptoms of term and pre-term PE, as well as in the clinical phase of PE at term. The specific geometric pattern strongly associated with PE was characterized by the presence of a basal septal bulge (SB), which was reported in women with PE at term exhibiting signs of global diastolic dysfunction. SB was also found in normotensive women at risk, who subsequently developed pre-term PE, but it was absent in women with uncomplicated pregnancies.

SB seems to be a specific, localized structural change in the myocardium, which develops in response to elevated after-load and helps maintain the balance between the oxygen demand and supply of the heart muscle [92].

This specific pattern of LV remodeling was also described in a prospective study, which used MRI to assess the cardiac structure in women with PE [93]. MRI assessment was clinically valuable in risk stratification and was suggested as an alternative method for analyzing the function of the maternal cardiac system, as it is safe and acceptable for pregnant women. One advantage of this examination method is that MRI does not require extensive expertise and special training of medical staff to perform the analysis and obtain reliable results, unlike echocardiography.

#### 4.2.4. Echocardiography Assessment Can Improve Screening for Pre-Eclampsia

In order to find another good predictor of PE, Garcia Gonzales et al. [94] assessed cardiac indices in two groups of women at 35 + 0 to 36 + 6 weeks of gestation: those who developed PE (50) and those with uncomplicated pregnancies (1552). Women later diagnosed with PE—even without symptoms of PE at the time of assessment—exhibited significantly increased left ventricle mass index (LVMI) in terms of body surface area and less favorable LV diastolic indices compared to women with uneventful pregnancies. The increase in the ratio between early mitral inflow velocity and mitral annular early diastolic velocity (E/e’)—a parameter directly related to left ventricle filling pressure and LVMI—had been previously reported in healthy women during the third trimester of gestation, probably as a manifestation of the mother’s CVS physiological adaptive response [85].

The important finding of this study was the establishment of threshold values for E/e’ and LMVI, which improved the prediction of PE at term.

The addition of cardiovascular assessment to the screening test for PE may help improve the stratification of women at risk of developing PE. However, further studies are required to determine the most accurate technique and timing during pregnancy for performing these examinations.

#### 4.2.5. Early PE and Late PE—Two Types of Disease vs. Two Different Disease Entities

Changes in the structure and function of the maternal heart differ not only between healthy pregnant women and those with hypertensive disorders but also between specified subtypes of PE.

Valensise et al. [95] conducted a study to compare the maternal cardiac function at 24 weeks of gestation in normotensive women who were later diagnosed with PE (early or late) to identify the potential differences specific to each subtype of the disease. Late PE exhibited the highest LVMI and CO values and the lowest TVR compared to early PE and the controls. In addition to the differences in cardiac indices, differences in maternal characteristics were identified. Early PE was associated with a higher percentage of patients over 35 years of age, a higher incidence of bilateral notching in UA Doppler at mid-gestation, lower gestational age at the time of delivery and lower neonatal weight compared to late PE and the controls. Women who developed late PE had higher pre-conception BMI compared to those with early PE and the controls.

This finding partially contrasts with the results obtained by Melchiorre et al. [96], who also found a high TVR/low CO profile in women who subsequently developed pre-term PE. However, women with later diagnosis of term PE exhibited high values of TVR and unchanged CO.

This inconsistency can be attributed to the fact that, in the latter study, CO was adjusted to the body surface area, and the hemodynamic profile was presented using the cardiac index (CI) parameter.

The results of these studies led to identification of two hemodynamic profiles preceding the onset of PE. The first profile is characterized by high TVR and low cardiac output (features often associated with co-occurrence of FGR) and predisposes to the development of early PE. The other hemodynamic state is characterized by low TVR and high or unchanged CO and is highly prognostic of late PE.

#### 4.2.6. Hemodynamic Profile in Pre-Eclampsia Depends on the Co-Occurrence of FGR

Stott et al. [97] also investigated hemodynamic profiles in a group of women at high risk of developing PE, but in this study, they stratified PE by the presence or absence of FGR. The main finding, which emerged from this research, was that the cardiac output was lower in women diagnosed with both PE and FGR compared to those with PE only and the control group of normotensive healthy women. The occurrence of PE was associated with high values of peripheral vascular resistance and mean arterial pressure—independent of the diagnosis of FGR—compared to the controls.

This is partly consistent with a study performed by Tay et al. [98], which showed that women with PE had a higher cardiac output and lower peripheral vascular resistance compared to a control group of uncomplicated pregnancies. However, those with both PE and FGR demonstrated lower CO and higher PVR. These changes occurred regardless of the gestational age at onset. Moreover, low cardiac output was associated with low birth weight, and high cardiac output was associated with high birth weight.

Another study indicated that anti-hypertensive therapy in pregnant women at risk of PE aimed at avoiding the suppression of CO resulted in preserved fetal growth. This suggests that the proper functioning of the maternal cardiovascular system ensures normal fetal growth not only at the time of placentation but throughout the pregnancy as well. It can be concluded that iatrogenic interventions, which impact maternal hemodynamics, can also influence the condition of the fetus, emphasizing the importance of the introduction of a well-selected treatment at the right time to avoid certain pregnancy-related complications [99].

#### 4.2.7. The Effects of PE Extend beyond Maternal–Fetal Circulation

It is worth noting that PE not only impacts the part of the circulatory system responsible for ensuring optimal uteroplacental flow, but it also has an impact on cerebral circulation.

A prospective observational study involving 2287 pregnancies at 35–37 weeks of gestation—with 60 (2.6%) women who developed PE—showed that maternal ophthalmic artery Doppler can be useful in predicting the subsequent development of PE and gestational hypertension [100]. This finding is consistent with previous cross-sectional studies, which reported characteristic waveform abnormalities in women with PE compared to normotensive pregnant women. However, the most effective prediction of PE can be achieved by combining maternal demographic characteristics and medical history with biomarkers such as the uterine artery pulsatility index (UtA-PI), mean arterial pressure (MAP), serum placental growth factor (PlGF) and serum soluble fms-like tyrosine kinase-1 (sFlt-1) [101].

### 4.3. PE Is Associated with Long-Term Postpartum Cardiovascular Complications

#### 4.3.1. The Proper Adaptation of a Woman’s Body to Pregnancy Is Important for Avoiding Pregnancy Complications

Referring to the theory of defective placentation, a belief has prevailed that birth is “a cure for PE” and that all negative effects of the disease on mother’s health are completely reversible upon the delivery of a child and placenta. However, numerous studies have proved that PE has lasting adverse effects on women’s health not only during pregnancy but for a long time after delivery.

In a healthy pregnancy, the maternal cardiovascular system has to undergo the necessary adaptations to accommodate the increased volume load by generating a remodeling response, which principally affect the LV [102]. To understand the significance of these changes, we can compare the approximately 35% increase in left ventricle mass reported in pregnant women with ≈ a 25% increase in LMV observed in athletes after one year of intensive training [103].

Pregnancy can therefore be considered a form of training of the cardiovascular system, resulting in improved cardiac adaptation in subsequent pregnancies and a reduced risk of developing PE. This notion is supported by several studies highlighting the advantage of cardiac adaptation in multiparous women compared to nulliparous women.

#### 4.3.2. Beneficial Effect of Multiparity

An echocardiographic study performed by Turan et al. [104] found that left ventricular systolic function in the first trimester of physiological pregnancy in primiparous women differs from that found in multiparous women. Multiparous women exhibited a more favorable cardiovascular profile, characterized by higher cardiac output, cardiac index, lower mean arterial pressure and peripheral resistance. Furthermore, the differences in these cardiovascular parameters between multiparae and nulliparae increased with parity.

This finding is consistent with a prospective study performed by Ling et al. [105], which investigated the impact of parity on maternal hemodynamics. The study included three groups of women (parous women without a history of PE and/or small for gestational age (SGA), nulliparous women and parous women with a history of PE and/or SGA). Throughout gestation, these women were assessed multiple times. The results showed that multiparous women had superior cardiac adaptation, reflected in the higher cardiac output and lower peripheral vascular resistance. In contrast, multiparous women with a history of PE and/or SGA displayed lower cardiac output and higher peripheral vascular resistance from mid-gestation.

The findings from these studies suggest that the cardiovascular system in women during their first pregnancy adapts to the increased workload. This adaptation leads to more effective mobilization of cardiovascular adaptive mechanisms in subsequent pregnancies, thereby improving the cardiovascular response. This kind of “training” has a positive impact on the process of placentation and consequently reduces the risk of developing PE.

#### 4.3.3. The Occurrence of PE Predisposes to Its Recurrence in Subsequent Pregnancies

On the other hand, a history of PE in a previous pregnancy is a risk factor for its recurrence in subsequent pregnancies. This correlation proves that PE exerts a prolonged impact on the condition of the cardiovascular system, long after the pregnancy affected by this disease has ended. This is consistent with the observation that the cardiovascular profile before conception in women with recurrent PE differs from that of women who did not experience PE in their subsequent pregnancies [82]. This suggests that the cardiovascular system in certain women who had PE did not fully recover from the damage caused by the disease in their previous pregnancy. These findings prompted the scientists to further investigate the function of CVS in postpartum women who experienced hypertensive disorders, particularly PE.

#### 4.3.4. Persistence of Changes in the Structure and Function of the Heart Muscle

It has been proved that the alterations in the function and structure of the left ventricle, which occur during pregnancies affected by PE, do not regress completely after delivery in a significant number of cases. A prospective longitudinal study by Melchiorre et al. [106] aimed to assess whether the abnormalities observed in women with PE persist after giving birth. Echocardiography was performed within 24 h of the diagnosis of PE, as well as 1 and 2 years after delivery in women diagnosed with PE and in healthy normotensive controls. The study found that nearly half of the women diagnosed with pre-term PE showed persistent global LV dysfunction during follow-up assessments. Although the altered LV geometry seen during the acute phase of PE mostly returned to its pre-pregnancy state in both pre-term and term PE cases, some pre-term subjects still exhibited abnormal LV geometry in the postpartum period. Additionally, the ratio of LV mass to cardiac work ratio and LV wall stress were significantly higher in pre-term PE compared to term PE and the controls. The presence of structural and functional changes in the maternal cardiovascular system detectable after delivery was more pronounced in women with pre-term PE compared to those with term PE and the control group. Furthermore, women with pre-term PE were at a higher risk of developing essential hypertension within 1–2 years following delivery compared to the term PE group and the control group. Other studies supported these findings by identifying geometric alterations and functional impairments of the LV, although these evaluations were performed solely in postpartum women with PE [107].

#### 4.3.5. The Impact of PE on Women’s Health Later in Life

A history of PE continues to affect women’s health in the postpartum period and later in life. The persistence of subclinical features of cardiac dysfunction is strongly associated with cardiovascular morbidity and mortality. Women with a history of PE/eclampsia have an increased risk of developing diabetes, dyslipidemia, hypertension, congestive heart failure and cerebrovascular disease in the future [108]. This correlation between the occurrence of CVD later in life and a history of PE is particularly pronounced in cases of severe PE, early onset of hypertensive disorders, the co-occurrence of FGR, recurrence of hypertensive complications in subsequent pregnancy and the need for pre-term delivery due to disease progression [109].

The role of PE in the development of cardiovascular complications is so significant that the guidelines for preventing CVD established by the American Heart Association have included a history of PE in the algorithm for evaluating the 10-year Framingham cardiovascular risk score [66].

In order to mitigate the potentially serious health consequences for women who have experienced PE, targeted screening examinations—possibly based on cardiac assessment—should be implemented. Obstetricians must extend efforts to raise awareness among women about their elevated risk of cardiovascular events compared to the general population. In the management of high-risk patients, physicians should focus on lifestyle modification (weight control, a healthy diet, regular exercise) and consider the early initiation of anti-hypertensive treatment, preferably adjusted to the specific cardiovascular profile of the patient.

## 5. Conclusions

Although the two-stage model of PE is the most popular theory of the disease, the cardiac origin of PE must also be taken into consideration as a potential pathomechanism. A common element in both theories is impaired placental perfusion. It is possible that primary impaired placentation—as well as a reduced perfusion due to cardiac and cardiovascular system maladaptation and malfunction—may predispose to angiogenic imbalance, a phenomenon characteristic of PE.

Awareness of the extreme heterogeneity of PE in the context of complicated and distinct pathomechanisms of the disease should encourage clinicians to focus on different phenotypes of women as candidates for the development of PE. At the same time, this review emphasizes the need for interdisciplinary management of PE, as well as possible variations in treatment options based on an individual approach to each patient.

## Data Availability

Not applicable.

## References

[1] Yang Y., Le Ray I., Zhu J., Zhang J., Hua J., Reilly M. (2021). Preeclampsia Prevalence, Risk Factors, and Pregnancy Outcomes in Sweden and China. JAMA Netw. Open.

[2] Zhang M., Wan P., Ng K., Singh K., Cheng T.H., Velickovic I., Dalloul M., Wlody D. (2020). Preeclampsia Among African American Pregnant Women: An Update on Prevalence, Complications, Etiology, and Biomarkers. Obstet. Gynecol. Surv..

[3] Rolnik D.L., Wright D., Poon L.C.Y., Syngelaki A., O’Gorman N., de Paco Matallana C., Akolekar R., Cicero S., Janga D., Singh M. (2020). ASPRE trial: Performance of screening for preterm pre-eclampsia. Ultrasound Obstet. Gynecol..

[4] Kornacki J., Wender-Ożegowska E. (2020). Utility of biochemical tests in prediction, diagnostics and clinical management of preeclampsia: A review. Arch. Med. Sci. AMS.

[5] Kornacki J., Wirstlein P., Wender-Ozegowska E. (2019). Levels of syndecan-1 and hyaluronan in early- and late-onset preeclampsia. Pregnancy Hypertens..

[6] Kornacki J., Gutaj P., Kalantarova A., Sibiak R., Jankowski M., Wender-Ozegowska E. (2021). Endothelial Dysfunction in Pregnancy Complications. Biomedicines.

[7] Karumanchi S.A. (2016). Angiogenic Factors in Preeclampsia: From Diagnosis to Therapy. Hypertension.

[8] Kalafat E., Thilaganathan B. (2017). Cardiovascular origins of preeclampsia. Curr. Opin. Obstet. Gynecol..

[9] Redman C.W. (1991). Current topic: Pre-eclampsia and the placenta. Placenta.

[10] Staff A.C. (2019). The two-stage placental model of preeclampsia: An update. J. Reprod. Immunol..

[11] Huppertz B. (2019). Traditional and New Routes of Trophoblast Invasion and Their Implications for Pregnancy Diseases. Int. J. Mol. Sci..

[12] Staff A.C., Fjeldstad H.E., Fosheim I.K., Moe K., Turowski G., Johnsen G.M., Alnaes-Katjavivi P., Sugulle M. (2022). Failure of physiological transformation and spiral artery atherosis: Their roles in preeclampsia. Am. J. Obstet. Gynecol..

[13] Pijnenborg R., Vercruysse L., Hanssens M. (2006). The uterine spiral arteries in human pregnancy: Facts and controversies. Placenta.

[14] Burton G.J., Charnock-Jones D.S., Jauniaux E. (2009). Regulation of vascular growth and function in the human placenta. Reproduction.

[15] Harris L.K. (2011). IFPA Gabor Than Award lecture: Transformation of the spiral arteries in human pregnancy: Key events in the remodelling timeline. Placenta.

[16] Robertson W.B., Brosens I., Dixon H.G. (1967). The pathological response of the vessels of the placental bed to hypertensive pregnancy. J. Pathol. Bacteriol..

[17] Kornacki J., Skrzypczak J. (2008). Preeclampsia-two manifestations of the same disease. Ginekol. Polska.

[18] Burton G.J., Woods A.W., Jauniaux E., Kingdom J.C. (2009). Rheological and physiological consequences of conversion of the maternal spiral arteries for uteroplacental blood flow during human pregnancy. Placenta.

[19] Burton G.J., Yung H.W., Cindrova-Davies T., Charnock-Jones D.S. (2009). Placental endoplasmic reticulum stress and oxidative stress in the pathophysiology of unexplained intrauterine growth restriction and early onset preeclampsia. Placenta.

[20] Ding J., Zhang Y., Cai X., Diao L., Yang C., Yang J. (2021). Crosstalk Between Trophoblast and Macrophage at the Maternal-Fetal Interface: Current Status and Future Perspectives. Front. Immunol..

[21] Staff A.C., Johnsen G.M., Dechend R., Redman C.W.G. (2014). Preeclampsia and uteroplacental acute atherosis: Immune and inflammatory factors. J. Reprod. Immunol..

[22] Robson A., Harris L.K., Innes B.A., Lash G.E., Aljunaidy M.M., Aplin J.D., Baker P.N., Robson S.C., Bulmer J.N. (2012). Uterine natural killer cells initiate spiral artery remodeling in human pregnancy. FASEB J..

[23] Tilburgs T., Scherjon S.A., van der Mast B.J., Haasnoot G.W., Versteeg V.D., Voort-Maarschalk M., Roelen D.L., van Rood J.J., Claas F.H. (2009). Fetal-maternal HLA-C mismatch is associated with decidual T cell activation and induction of functional T regulatory cells. J. Reprod. Immunol..

[24] Robertson S.A., Care A.S., Moldenhauer L.M. (2018). Regulatory T cells in embryo implantation and the immune response to pregnancy. J. Clin. Investig..

[25] Robinson E.S., Khankin E.V., Choueiri T.K., Dhawan M.S., Rogers M.J., Karumanchi S.A., Humphreys B.D. (2010). Suppression of the nitric oxide pathway in metastatic renal cell carcinoma patients receiving vascular endothelial growth factor-signaling inhibitors. Hypertension.

[26] Saleh L., Danser J.A., van den Meiracker A.H. (2016). Role of endothelin in preeclampsia and hypertension following antiangiogenesis treatment. Curr. Opin. Nephrol. Hypertens..

[27] Meekins J.W., Pijnenborg R., Hanssens M., van Assche A., McFadyen I.R. (1994). Immunohistochemical detection of lipoprotein(a) in the wall of placental bed spiral arteries in normal and severe preeclamptic pregnancies. Placenta.

[28] Robertson W.B., Brosens I., Dixon G. (1975). Uteroplacental vascular pathology. Eur. J. Obstet. Gynecol. Reprod. Biol..

[29] Staff A.C., Dechend R., Pijnenborg R. (2010). Learning from the placenta: Acute atherosis and vascular remodeling in preeclampsia-novel aspects for atherosclerosis and future cardiovascular health. Hypertension.

[30] Brosens I. (1996). How the role of the spiral arteries in the pathogenesis of preeclampsia was discovered. Hypertens. Pregnancy.

[31] Kornacki J., Wirstlein P., Skrzypczak J. (2013). Concentrations of antiangiogenic factors, triglycerides, glucose and insulin in women with two types of preeclampsia. Ginekol. Polska.

[32] Boroń D., Kornacki J., Gutaj P., Mantaj U., Wirstlein P., Wender-Ozegowska E. (2022). Corin-the Early Marker of Preeclampsia in Pregestational Diabetes Mellitus. J. Clin. Med..

[33] Kornacki J., Wirstlein P., Wender-Ożegowska E. (2020). Markers of Endothelial Injury and Dysfunction in Early- and Late-Onset Preeclampsia. Life.

[34] Young B.C., Levine R.J., Karumanchi S.A. (2010). Pathogenesis of preeclampsia. Annu. Rev. Pathol..

[35] Roberts J.M., Rajakumar A. (2009). Preeclampsia and soluble fms-like tyrosine kinase 1. J. Clin. Endocrinol. Metab..

[36] Zhou Y., McMaster M., Woo K., Janatpour M., Perry J., Karpanen T., Alitalo K., Damsky C., Fisher S.J. (2002). Vascular endothelial growth factor ligands and receptors that regulate human cytotrophoblast survival are dysregulated in severe preeclampsia and hemolysis, elevated liver enzymes, and low platelets syndrome. Am. J. Pathol..

[37] Jena M.K., Sharma N.R., Petitt M., Maulik D., Nayak N.R. (2020). Pathogenesis of Preeclampsia and Therapeutic Approaches Targeting the Placenta. Biomolecules.

[38] Boeckel J.N., Guarani V., Koyanagi M., Roexe T., Lengeling A., Schermuly R.T., Gellert P., Braun T., Zeiher A., Dimmeler S. (2011). Jumonji domain-containing protein 6 (Jmjd6) is required for angiogenic sprouting and regulates splicing of VEGF-receptor 1. Proc. Natl. Acad. Sci. USA.

[39] Palmer K.R., Tong S., Tuohey L., Cannon P., Ye L., Hannan N.J., Brownfoot F.C., Illanes S.E., Kaitu’u-Lino T.J. (2016). Jumonji Domain Containing Protein 6 Is Decreased in Human Preeclamptic Placentas and Regulates sFLT-1 Splice Variant Production. Biol. Reprod..

[40] Phipps E.A., Thadhani R., Benzing T., Karumanchi S.A. (2019). Pre-eclampsia: Pathogenesis, novel diagnostics and therapies. Nat. Rev. Nephrol..

[41] Venkatesha S., Toporsian M., Lam C., Hanai J., Mammoto T., Kim Y.M., Bdolah Y., Lim K.H., Yuan H.T., Libermann T.A. (2006). Soluble endoglin contributes to the pathogenesis of preeclampsia. Nat. Med..

[42] Levine R.J., Maynard S.E., Qian C., Lim K.H., England L.J., Yu K.F., Schisterman E.F., Thadhani R., Sachs B.P., Epstein F.H. (2004). Circulating angiogenic factors and the risk of preeclampsia. N. Engl. J. Med..

[43] Zachary I., Mathur A., Yla-Herttuala S., Martin J. (2000). Vascular protection: A novel nonangiogenic cardiovascular role for vascular endothelial growth factor. Arterioscler. Thromb. Vasc. Biol..

[44] Laresgoiti-Servitje E. (2013). A leading role for the immune system in the pathophysiology of preeclampsia. J. Leukoc. Biol..

[45] Chaiworapongsa T., Chaemsaithong P., Yeo L., Romero R. (2014). Pre-eclampsia part 1: Current understanding of its pathophysiology. Nat. Rev. Nephrol..

[46] McElwain C.J., Tuboly E., McCarthy F.P., McCarthy C.M. (2020). Mechanisms of Endothelial Dysfunction in Pre-eclampsia and Gestational Diabetes Mellitus: Windows into Future Cardiometabolic Health?. Front. Endocrinol..

[47] Mannaerts D., Faes E., Goovaerts I., Stoop T., Cornette J., Gyselaers W., Spaanderman M., Van Craenenbroeck E.M., Jacquemyn Y. (2017). Flow-mediated dilation and peripheral arterial tonometry are disturbed in preeclampsia and reflect different aspects of endothelial function. Am. J. Physiol. Regul. Integr. Comp. Physiol..

[48] Musz P., Podhajski P., Grzelakowska K., Uminska J.M. (2021). Non-Invasive Assessment of Endothelial Function—A Review of Available Methods. Med. Res. J..

[49] Meeme A., Buga G.A., Mammen M., Namugowa A. (2017). Endothelial dysfunction and arterial stiffness in pre-eclampsia demonstrated by the EndoPAT method. Cardiovasc. J. Afr..

[50] Vural P. (2002). Nitric oxide/endothelin-1 in preeclampsia. Clin. Chim. Acta Int. J. Clin. Chem..

[51] Baksu B., Davas I., Baksu A., Akyol A., Gulbaba G. (2005). Plasma nitric oxide, endothelin-1 and urinary nitric oxide and cyclic guanosine monophosphate levels in hypertensive pregnant women. Int. J. Gynaecol. Obstet..

[52] Bernardi F., Constantino L., Machado R., Petronilho F., Dal-Pizzol F. (2008). Plasma nitric oxide, endothelin-1, arginase and superoxide dismutase in pre-eclamptic women. J. Obstet. Gynaecol. Res..

[53] Aydin S., Benian A., Madazli R., Uludag S., Uzun H., Kaya S. (2004). Plasma malondialdehyde, superoxide dismutase, sE-selectin, fibronectin, endothelin-1 and nitric oxide levels in women with preeclampsia. Eur. J. Obstet. Gynecol. Reprod. Biol..

[54] Docheva N., Romero R., Chaemsaithong P., Tarca A.L., Bhatti G., Pacora P., Panaitescu B., Chaiyasit N., Chaiworapongsa T., Maymon E. (2019). The profiles of soluble adhesion molecules in the “great obstetrical syndromes”. J. Matern. Neonatal Med..

[55] Mistry H.D., Ogalde M.V.H., Broughton Pipkin F., Escher G., Kurlak L.O. (2020). Maternal, Fetal, and Placental Selectins in Women With Pre-eclampsia; Association With the Renin-Angiotensin-System. Front. Med..

[56] Papakonstantinou K., Economou E., Koupa E., Babameto I., Hasiakos D., Vitoratos N. (2011). Antepartum and postpartum maternal plasma levels of E-selectin and VE-cadherin in preeclampsia, gestational proteinuria and gestational hypertension. J. Matern. Neonatal Med..

[57] Hassan H.E., Azzam H., Othman M., Hassan M., Selim T. (2019). Soluble E-selectin, platelet count and mean platelet volume as biomarkers for pre-eclampsia. Pregnancy Hypertens..

[58] Mehrabian F., Jazi S.M., Javanmard S.H., Kaviani M., Homayouni V. (2012). Circulating endothelial cells (CECs) and E-selectin: Predictors of preeclampsia. J. Res. Med. Sci. Off. J. Isfahan Univ. Med. Sci..

[59] Szpera-Goździewicz A., Majcherek M., Boruczkowski M., Goździewicz T., Dworacki G., Wicherek L., Bręborowicz G.H. (2017). Circulating endothelial cells, circulating endothelial progenitor cells, and von Willebrand factor in pregnancies complicated by hypertensive disorders. Am. J. Reprod. Immunol..

[60] Anim-Nyame N., Ghosh A., Freestone N., Arrigoni F.I. (2015). Relationship between insulin resistance and circulating endothelial cells in pre-eclampsia. Gynecol. Endocrinol..

[61] Rios D.R.A., Alpoim P.N., Godoi L.C., Perucci L.O., de Sousa L.P., Gomes K.B., Dusse L.M.S. (2016). Increased Levels of sENG and sVCAM-1 and Decreased Levels of VEGF in Severe Preeclampsia. Am. J. Hypertens..

[62] Weissgerber T.L., Garcia-Valencia O., Milic N.M., Codsi E., Cubro H., Nath M.C., White W.M., Nath K.A., Garovic V.D. (2019). Early Onset Preeclampsia Is Associated with Glycocalyx Degradation and Reduced Microvascular Perfusion. J. Am. Heart Assoc..

[63] Verlohren S., Brennecke S.P., Galindo A., Karumanchi S.A., Mirkovic L.B., Schlembach D., Stepan H., Vatish M., Zeisler H., Rana S. (2022). Clinical interpretation and implementation of the sFlt-1/PlGF ratio in the prediction, diagnosis and management of preeclampsia. Pregnancy Hypertens..

[64] Wu P., Haththotuwa R., Kwok C.S., Babu A., Kotronias R.A., Rushton C., Zaman A., Fryer A.A., Kadam U., Chew-Graham C.A. (2017). Preeclampsia and Future Cardiovascular Health: A Systematic Review and Meta-Analysis. Circulation. Cardiovasc. Qual. Outcomes.

[65] Espinoza J., Vidaeff A., Pettker C.M., Simhan H. (2020). Gestational Hypertension and Preeclampsia: ACOG Practice Bulletin, Number 222. Obstet. Gynecol..

[66] Mosca L., Benjamin E.J., Berra K., Bezanson J.L., Dolor R.J., Lloyd-Jones D.M., Newby L.K., Piña I.L., Roger V.L., Shaw L.J. (2011). Effectiveness-based guidelines for the prevention of cardiovascular disease in women—2011 update: A guideline from the american heart association. Circulation.

[67] Bushnell C., McCullough L.D., Awad I.A., Chireau M.V., Fedder W.N., Furie K.L., Howard V.J., Lichtman J.H., Lisabeth L.D., Piña I.L. (2014). Guidelines for the prevention of stroke in women: A statement for healthcare professionals from the American Heart Association/American Stroke Association. Stroke.

[68] Giannakou K., Evangelou E., Papatheodorou S.I. (2018). Genetic and non-genetic risk factors for pre-eclampsia: Umbrella review of systematic reviews and meta-analyses of observational studies. Ultrasound Obstet. Gynecol..

[69] Palomba S., de Wilde M.A., Falbo A., Koster M.P., La Sala G.B., Fauser B.C. (2015). Pregnancy complications in women with polycystic ovary syndrome. Hum. Reprod. Update.

[70] Zhou M.S., Wang A., Yu H. (2014). Link between insulin resistance and hypertension: What is the evidence from evolutionary biology?. Diabetol. Metab. Syndr..

[71] Heida K.Y., Bots M.L., de Groot C.J., van Dunné F.M., Hammoud N.M., Hoek A., Laven J.S., Maas A.H., Roeters van Lennep J.E., Velthuis B.K. (2016). Cardiovascular risk management after reproductive and pregnancy-related disorders: A Dutch multidisciplinary evidence-based guideline. Eur. J. Prev. Cardiol..

[72] Serrano N.C., Guio-Mahecha E., Quintero-Lesmes D.C., Becerra-Bayona S., Paez M.C., Beltran M., Herrera V.M., Leon L.J., Williams D., Casas J.P. (2018). Lipid profile, plasma apolipoproteins, and pre-eclampsia risk in the GenPE case-control study. Atherosclerosis.

[73] Chen Z., Chen G., Qin H., Cai Z., Huang J., Chen H., Wu W., Chen Z., Wu S., Chen Y. (2020). Higher triglyceride to high-density lipoprotein cholesterol ratio increases cardiovascular risk: 10-year prospective study in a cohort of Chinese adults. J. Diabetes Investig..

[74] Marais A.D. (2019). Apolipoprotein E in lipoprotein metabolism, health and cardiovascular disease. Pathology.

[75] Walldius G. (2012). The apoB/apoA-I Ratio is a Strong Predictor of Cardiovascular Risk. Lipoproteins—Role in Health and Diseases.

[76] Romundstad P.R., Magnussen E.B., Smith G.D., Vatten L.J. (2010). Hypertension in pregnancy and later cardiovascular risk: Common antecedents?. Circulation.

[77] Egeland G.M., Klungsøyr K., Øyen N., Tell G.S., Næss Ø., Skjærven R. (2016). Preconception Cardiovascular Risk Factor Differences Between Gestational Hypertension and Preeclampsia: Cohort Norway Study. Hypertension.

[78] England L., Zhang J. (2007). Smoking and risk of preeclampsia: A systematic review. Front. Biosci..

[79] Office of the Surgeon General (US), Office on Smoking and Health (US) (2004). The Health Consequences of Smoking: A Report of the Surgeon General.

[80] Sattar N., Greer I.A. (2002). Pregnancy complications and maternal cardiovascular risk: Opportunities for intervention and screening?. BMJ.

[81] Sep S.J., Schreurs M.P., Bekkers S.C., Kruse A.J., Smits L.J., Peeters L.L. (2011). Early-pregnancy changes in cardiac diastolic function in women with recurrent pre-eclampsia and in previously pre-eclamptic women without recurrent disease. BJOG Int. J. Obstet. Gynaecol..

[82] Ghossein-Doha C., Spaanderman M.E., Al Doulah R., Van Kuijk S.M., Peeters L.L. (2016). Maternal cardiac adaptation to subsequent pregnancy in formerly pre-eclamptic women according to recurrence of pre-eclampsia. Ultrasound Obstet. Gynecol..

[83] Longo L.D. (1983). Maternal blood volume and cardiac output during pregnancy: A hypothesis of endocrinologic control. Am. J. Physiol..

[84] Mahendru A.A., Everett T.R., Wilkinson I.B., Lees C.C., McEniery C.M. (2014). A longitudinal study of maternal cardiovascular function from preconception to the postpartum period. J. Hypertens..

[85] Melchiorre K., Sharma R., Khalil A., Thilaganathan B. (2016). Maternal Cardiovascular Function in Normal Pregnancy: Evidence of Maladaptation to Chronic Volume Overload. Hypertension.

[86] Ferreira A.F., Azevedo M.J., Proenca T., Saraiva F.A., Machado A.P., Sousa C., Sampaio Maia B., Leite-Moreira A., Ramalho C., Fa I. (2021). Cardiac remodelling and reverse remodelling in pregnant women: What can be expected?. Eur. Heart J..

[87] Hayward R.M., Foster E., Tseng Z.H. (2017). Maternal and Fetal Outcomes of Admission for Delivery in Women with Congenital Heart Disease. JAMA Cardiol..

[88] Drenthen W., Boersma E., Balci A., Moons P., Roos-Hesselink J.W., Mulder B.J., Vliegen H.W., van Dijk A.P., Voors A.A., Yap S.C. (2010). Predictors of pregnancy complications in women with congenital heart disease. Eur. Heart J..

[89] Yap S.C., Drenthen W., Meijboom F.J., Moons P., Mulder B.J., Vliegen H.W., van Dijk A.P., Jaddoe V.W., Steegers E.A., Roos-Hesselink J.W. (2009). Comparison of pregnancy outcomes in women with repaired versus unrepaired atrial septal defect. BJOG Int. J. Obstet. Gynaecol..

[90] Vasapollo B., Novelli G.P., Valensise H. (2008). Total vascular resistance and left ventricular morphology as screening tools for complications in pregnancy. Hypertension.

[91] Melchiorre K., Sutherland G.R., Baltabaeva A., Liberati M., Thilaganathan B. (2011). Maternal cardiac dysfunction and remodeling in women with preeclampsia at term. Hypertension.

[92] Gaudron P.D., Liu D., Scholz F., Hu K., Florescu C., Herrmann S., Bijnens B., Ertl G., Störk S., Weidemann F. (2016). The septal bulge—An early echocardiographic sign in hypertensive heart disease. J. Am. Soc. Hypertens. JASH.

[93] Hall M., de Marvao A., Schweitzer R., Cromb D., Colford K., Jandu P., O’Regan D.P., Ho A., Price A., Chappell L.C. (2023). Characterisation of placental, fetal brain and maternal cardiac structure and function in pre-eclampsia using MRI. arXiv.

[94] Garcia-Gonzalez C., Georgiopoulos G., Azim S.A., Macaya F., Kametas N., Nihoyannopoulos P., Nicolaides K.H., Charakida M. (2020). Maternal Cardiac Assessment at 35 to 37 Weeks Improves Prediction of Development of Preeclampsia. Hypertension.

[95] Valensise H., Vasapollo B., Gagliardi G., Novelli G.P. (2008). Early and late preeclampsia: Two different maternal hemodynamic states in the latent phase of the disease. Hypertension.

[96] Melchiorre K., Sutherland G., Sharma R., Nanni M., Thilaganathan B. (2013). Mid-gestational maternal cardiovascular profile in preterm and term pre-eclampsia: A prospective study. BJOG Int. J. Obstet. Gynaecol..

[97] Stott D., Nzelu O., Nicolaides K.H., Kametas N.A. (2018). Maternal hemodynamics in normal pregnancy and in pregnancy affected by pre-eclampsia. Ultrasound Obstet. Gynecol..

[98] Tay J., Foo L., Masini G., Bennett P.R., McEniery C.M., Wilkinson I.B., Lees C.C. (2018). Early and late preeclampsia are characterized by high cardiac output, but in the presence of fetal growth restriction, cardiac output is low: Insights from a prospective study. Am. J. Obstet. Gynecol..

[99] Easterling T.R., Carr D.B., Brateng D., Diederichs C., Schmucker B. (2001). Treatment of hypertension in pregnancy: Effect of atenolol on maternal disease, preterm delivery, and fetal growth. Obstet. Gynecol..

[100] Sarno M., Wright A., Vieira N., Sapantzoglou I., Charakida M., Nicolaides K.H. (2020). Ophthalmic artery Doppler in prediction of pre-eclampsia at 35–37 weeks’ gestation. Ultrasound Obstet. Gynecol..

[101] Nicolaides K.H., Sarno M., Wright A. (2022). Ophthalmic artery Doppler in the prediction of preeclampsia. Am. J. Obstet. Gynecol..

[102] Melchiorre K., Sharma R., Thilaganathan B. (2012). Cardiac structure and function in normal pregnancy. Curr. Opin. Obstet. Gynecol..

[103] Arbab-Zadeh A., Perhonen M., Howden E., Peshock R.M., Zhang R., Adams-Huet B., Haykowsky M.J., Levine B.D. (2014). Cardiac remodeling in response to 1 year of intensive endurance training. Circulation.

[104] Turan O.M., De Paco C., Kametas N., Khaw A., Nicolaides K.H. (2008). Effect of parity on maternal cardiac function during the first trimester of pregnancy. Ultrasound Obstet. Gynecol..

[105] Ling H.Z., Guy G.P., Bisquera A., Poon L.C., Nicolaides K.H., Kametas N.A. (2019). The effect of parity on longitudinal maternal hemodynamics. Am. J. Obstet. Gynecol..

[106] Melchiorre K., Sutherland G.R., Liberati M., Thilaganathan B. (2011). Preeclampsia is associated with persistent postpartum cardiovascular impairment. Hypertension.

[107] Strobl I., Windbichler G., Strasak A., Weiskopf-Schwendinger V., Schweigmann U., Ramoni A., Scheier M. (2011). Left ventricular function many years after recovery from pre-eclampsia. BJOG Int. J. Obstet. Gynaecol..

[108] Kuo Y.L., Chan T.F., Wu C.Y., Ker C.R., Tu H.P. (2018). Preeclampsia-eclampsia and future cardiovascular risk among women in Taiwan. Taiwan. J. Obstet. Gynecol..

[109] Melchiorre K., Thilaganathan B., Giorgione V., Ridder A., Memmo A., Khalil A. (2020). Hypertensive Disorders of Pregnancy and Future Cardiovascular Health. Front. Cardiovasc. Med..

